# 

**Published:** 1833

**Authors:** 


### CONTENTS OF NO. XXV.

### Essays and Cases.

| Art. | | Page. |
|---|---|---|
| I. | Observations on Scarlet Fever. By Dr. Silas Reed, | 8 |
| II. | Remarks on Scarlet Fever. By George E. Conant, | 23 |
| III. | Notes of a Case in which Hydrophobia was prevented. By Landon C. Rives, M. D. - - - - - - | 27 |
| IV. | Observations on some of the uses and abuses of Purgatives. By Daniel Drake, M. D. - - - - - - | 32 |

### REVIEWS AND BIBLIOGRAPHICAL NOTICES.

| | | |
|---|---|---|
| V. | Epidemic Cholera: An Eclectic, Miscellaneous and Clinical Review, - - - - - - - - | 43 |
| | Mr. James Kennedy, - - - - - - | 44 |
| | Doctor Thomas Spencer, - - - - - | 50 |
| | —— Martin Paine, - - - - - - | 58 |
| | Mr. J. F. Fergus, - - - - - - | 61 |
| | Doctor John F. Henry, - - - - - - | 66 |
| | —— Yates, - - - - - - - | 70 |
| | —— Sturm, - - - - - - - | 71 |
| | —— Stevens, - - - - - - - | 72 |
| | —— Jackson, - - - - - - - | 78 |
| | —— Benjamin W. Dudley, - - - - | 87 |
| | In Lexington, - - - - - - - | 90 |
| | In Kentucky, - - - - - - - | 93 |
| | In Ohio—Contagion, - - - - - - | 96 |
| | In Cincinnati, - - - - - - - | 98 |

### MISCELLANEOUS INTELLIGENCE.

### I. Analectic.

1. Professor Ehrenberg's discoveries relative to the structure and functions of the Infusoria, 105.—2. Physiological investigations arising from the mechanical effects of pressure on the animal frame, 116.—3. Quantity of food taken by a person in health compared with the quantity of the different secretions during the same time, 120.—4. Experiments on Hemlock and Henbane, 122.—5. Cold Dash in Convulsions of Infants, 123.—6. On the Excisions of Hæmorrhoidal Tumours, 123.—7. Treatment of Prolapsus Ani, 131.—8. Treatment of Cholera by Cold, as practised by Dr. Casper, of Berlin, 133. —9. Dr. Hancock on External Stimulants, 135.—10. Clinical Observations on the Exhibition of Opium, in large doses, in certain cases of Disease. By Dr. W. Stokes, 139.—11. Dr. Broussais' Opinion on the use of Tartar Emetic in Pneumonia, 144.—12. Cholera, great variety in the Treatment of, in Paris, 145.

### II. Analytical.

1. Chemical and Medical Qualities of the Apocynum Cannabinum, 146.—2. Medicinal Properties of the Euphorbia Corollata, 147.—3. Spontaneous poisonous changes in Meat and Bread, 149.

### III. Original.

1. Our Enterprize, 151.—2. The Epidemic, 153.—3. Measles and Mumps in Combination, 154.—4. Convulsions in Infants, 155.—5. Anamalcules in our hydrant water, 155.—6. Neglect of Vaccination. Free Schools, 156.—7. Inquiry into the Condition of the Medical College of Ohio, 156.—8. Western Graduates, 157.—9. Popular Lectures on Anatomy and Surgery, 157.—10. Lithotomy, 158.—11. Medical Obituary, 158.—Dr. Roane, 158.—Dr. Pindell, 159.—Dr. Challen, 159.—Dr. Flanner, 159.—Dr. Richards, 159.—Dr. Heermann, 160.

---

### NO. XXVI.

### ESSAYS AND CASES.

| Art. | | Page. |
|---|---|---|
| I. | Epidemic Cholera: Miscellaneous Observations—historical, statistical, ætiological, and therapeutic, on the prevailing Epidemic. By Daniel Drake, M. D. | 161 |
| | 1. Spread of the Epidemic, | 162 |
| | 2. Ætiological Remarks, | 163 |
| | 3. Exciting Cause, | 168 |
| | 4. Treatment of the Epidemic in Lexington, | 169 |
| | 5. Cholera in Cincinnati, | 172 |
| | 6. Fatal Cases, | 174 |
| II. | Anomalous and Fatal Case of Disease. By A. W. Bowen, | 182 |

### REVIEWS AND BIBLIOGRAPHICAL NOTICES.

| | | |
|---|---|---|
| III. | Diseases of Louisiana. The application of Physiological Medicine to the Diseases of Louisiana. By Edward H. Barton, M. D. | 187 |
| IV. | Human Physiology. Human Physiology Illustrated by numerous Engravings. By Robley Dunglisson, M. D. | 193 |

V. Physical Education. Remarks on the influence of Mental Cultivation upon Health. By Amariah Brigham.
First Annual Report of the Society for promoting Manual Labor in Literary institutions, including a Report of their General Agent. By Theodore D. Weld, - - 226
1. Absurdities and Excesses in Diet, - - - 228
2. Abuse of Stimulating Drinks, - - - 229
3. Abuse of Tobacco, - - - - - - 230
4. Absurdities in Dress, . - - - - ib.
5. Irregularities in Sleep, - - - - - 232
6. Immersion in a Confined Atmosphere, - - ib.
7. Neglect of Exercise, - - - - - 233

### MISCELLANEOUS INTELLIGENCE.

### I. Analectic.

1. Observations on Cold as a cause of Disease, 256.—2. On Muscular Contractions, and Animal Electricity, 263.—3. Treatment of Inflammation of the Lungs, 265.—4. Gonorrhœa and its Treatment, 266.—5. The Anodyne Metallic or Galvanic Brush, 268.—6. Origin of Acephalocysts, 270.—7. Internal use of Chlorine in Nervous Fever, 271.—8. Endemic Therapeutics, 271.—9. Mode of Dressing a Stump after Amputation, 271.—10. Changes which the Points of the fingers undergo in Phthisis, 272.—11. Ingenious method of applying Nitras Argenti to the Ulcers of the Cornea, 273.—12. Roseolous Eruption, caused by the use of Copaiba and Cubebs, 273.—13. New Theory of the Sounds of the Heart, 274.—14. Ergot. By Dr. Borllcher, 274.—15. On Ergot, M. M. Trousseau, 275.—16. Chemical Analysis of Ergot, 276.—17. Anthelmintic Emulsion, 277.—18. Infantile Convulsions, 277.—19. Employment of Cloride of Lime in the Treatment of Psora, 281.—20. Chronic Bronchitis, 282.—22. Chloride of Soda in Burns, Scalds, and Black Eyes, 283.—23. Prolapsus Ani, 284.—24. On the use of Cold Water in Cholera, 286.—25. Use of Ice and Chloride of Soda in Cynanche Maligna, 290.—26. Efficacy of the Cold Dash in Nervous and Convulsive Diseases, 292.—27. On the Formation of Callous, and the mode of Remedying it when diseased or deformed. 293.—28. Gangrene of the Mouth in Children, 295.

### II. Analytical.

1. Puerperal Fever, 297.—2. Spinal Origin of Rheumatism, 305.—3. Electro Therapeutics, 309.—4. Dysmenorrhœa, 313.

### III. Original.

1. Our Second Hexade, 316.—2. Extra Professional Duties, 316.—3. Medical Journal on a New Plan, 318.—4. Geological Survey of the State of Tennessee, 319.—5. Flora of Kentucky, 319.—6. Cabinet of Entomology for sale, 319.—7. Mortification and Sloughing of a part of the Intestine in Hernia. Recovery without an External Aperture, 320.—8. Appointments in the Medical College of Ohio, 320.

### NO. XXVII.

### ESSAYS AND CASES.

| Art. | | Page. |
|---|---|---|
| I. | Epidemic Cholera in Yucatan. By Henry Perrine, M. D. | 321 |
| II. | Epidemic Cholera in Columbus, Ohio. By Wm. McClay Awl, M. D. - - - - - - - | 341 |
| III. | Spinal Irritation. By Charles C. Hildreth, M. D. - | 349 |
| IV. | Fistula of the Stomach. By Dr. John H. Cook, - | 353 |
| V. | Worms in the Bladder. By Dr. Leonard Bardwell, - | 354 |
| VI. | Geology of Ohio. By John L. Riddell, A. M. - | 356 |
| | Addendum to the same. By the Editor. - - | 364 |
| VII. | Shooting Stars. By the Editor. - - - - | 368 |

### REVIEWS AND BIBLIOGRAPHICAL NOTICES.

| | | |
|---|---|---|
| VIII. | American Cyclopædia of Practical Medicine and Surgery. Edited by Isaac Hays, M. D. - - - - | 386 |
| IX. | Imponderable Agents, - - - - - | 410 |

### MISCELLANEOUS INTELLIGENCE.

#### I. Analectic.

1. A Sketch of Broussais' Doctrines, drawn up by himself, 417.—2. Death of M. Portal, 421.—3. On Water, applied externally as a remedial agent, ib.—4. Ulceration of the Stomach, 425.—5. Doctor Graves on Peritonitis, 426.—6. Chemical Pathology of the Blood in Cholera, 428.—7. Treatment of Hooping Cough, 490.—8. Reproduction of the Crystalline Lens after the operation for Cataract, ib.—9. On the Bearing of Epidemics upon Medical Statistics and Political Economy, 431.—10. New and singular variety of Hermaphroditism, 433.—11. Extirpation of a degenerated Ovarium, 436.—12. Case of Hæmorrhage of the Uterus, arrested by compression of the descending Aorta, 437.—13. Professor Weber's experiments on the Sensibility of the Skin, 439.—15. Berzelius on the Chemical Constitution of Urine in various diseases, 444.—16. Observations on Local Blood-letting, and some new methods of practising it, 447.—17. On the Oil of the Croton Tiglium, as a Purgative for Children, 449.—18. Indian Ophthalmia, treated with success with Alum, 450.

#### II. Analytical.

1. Removal of the Clavicle in a state of Osteo-Sarcoma. By Prof. Warren, 451.—2. Injurious administration of the Sulphate of Quinine. By Dr. Monett, 452.—3. Effects of Lightning corrected by the Cold Affusion. By Dr. Young, 456.—4. Cholera cured by the Actea Racemosa. By the same, 557.—5. On Fractures of the Thigh and Leg. By Prof. Smith, of Baltimore, 558.—6. Admission of Air into the Veins, 563.

### III. Original.

1. Epidemic Cholera in the Peninsula of Yatucan, 565.—2. Bad Effects of Animal Diet, 556.—3. Remedy for Gleet, ib.—4. Open Foramen Ovale, 567.—5. Acephalous Fœtus, living eight hours, 568.—6. Poisoning by Oil of Tansey, 569.—7. Notice of a suddenly fatal disease, at Stamford, Kentucky, 570.—8. Permanent Paralysis of one side of face, from disease about the angle of the Jaw, 473.—9. Catalogue of Plants in Kentucky, 474.—10. Cultivation of Botany in Ohio, 476.—11. Cincinnati Medical Society, ib.—12. Report of the Commissioners appointed to inquire into the condition of the Medical College, and Commercial Hospital and Lunatic Asylum of Ohio, 477.—13. Repeal of the Medical Law of Ohio. Qualified approval. Suggestions about another Law, 479.—14. School for the Instruction of the Blind, 483.—15. Education Convention of Kentucky, 486.—16. Introduction of Tropical Plants into Florida, ib.—17. Winter residence for Consumptive Patients, 487.—18. Subsidence of the Cholera in the West. Health of Cincinnati, ib.—19. Medical Classes in the West for 1834, 488.—20. Medical Obituary, ib.

---

### NO. XXVIII.

### ESSAYS AND CASES.

| Art. | | Page. |
|---|---|---|
| I. | Irritation and Inflammation of the Peritoneum. By Daniel Drake, M. D. - - - - - - - - | 489 |
| 1. | Escape of the contents of the Stomach, - - - | 508 |
| 2. | Escape of the contents of the small Intestines, - - | 510 |
| 3. | Escape of the contents of the great Intestines, - - | 514 |
| 4. | Escape of the contents of the Gall Bladder, - - | 518 |
| 5. | Appendix, - - . - - - - - - | 524 |
| II. | Nitrate of Strichnine in Paralysis, in a Letter to the Editor, | 532 |
| III. | Cupping. By W. A. Gillespie, M. D. - - - | 534 |
| IV. | Operations for Calculi. By A. Hermange, M. D. - | 539 |

### REVIEWS AND BIBLIOGRAPHICAL NOTICES.

| | | |
|---|---|---|
| V. | Certainty of Medicine. By P. J. G. Cabinis. Translated from the French, by R. La Roche, M. D. - - - | 568 |

### MISCELLANEOUS INTELLIGENCE.

### I. Analectic.

1. On Hysteralgia, or irritable Uteras, 585.—2. Discharge of Worms from various parts of the body, 590.—3. Mr. Robertson on Dry Cupping, ib.—4. Use of Turpentine in Sciatic Neuralgias, 592.—

5. Lisfranc's Treatment of Amaurosis, ib.—6. Hydatids, and their Conversion into Tubercles, 593.—7. Chloride of Lime as a Lotion against the Itch, 594.—8. Dupuytren's Mode of Treating Prolapsus of the Rectum, ib.—9. Discovery of a new principle in the Serum of the Human Blood, ib.—10. Pathology and Treatment of Gastrites, 595.—11. Inflammation of the Bowels, 602.—12. Iodine in the Treatment of Salivation, ib.—13. Inflammation of the Mucous Membrane of the Bowels, 603.—14. On the Efficacy of Dry-Cupping in Various Diseases, 605.—15. On Obliteration of Veins, for the Cure of Varices, 610.—16. On the Presence of Copper in Wheat and several other substances, 611.—17. On the Transmission of Medicaments into the system by means of Electro Galvanism, 613.—18. Weight of Men at different Ages, 616.—19. Lunatics in New-York, 617.

### II. Original.

1. The Medical College of Ohio, 623.—Charges and Specifications against the Trustees, 625.—Charges and Specifications against the Faculty, 629.—Dr. Staughton's Letters and Evidence, 634.—Dr. Eberle's Letters, Extracts from, 637.—Remarks on the Report of the Senatorial Committee, 634.—Letters of Profs. Mitchell, Moorhead and Cobb, 647.—Remarks on the Correspondence, 649.—2. Popular Study of Anatomy and Physiology, 650.—3. Epidemic Cholera, 652.—4. Marine Hospitals on the Mississippi, ib.—5. Inquiries concerning Tubercular Diseases, 653.—6. Obituary notice, 655.—7. Meteorological Register, 656.—Specimens of Plants, 657.

### CONTENTS. OF NO. XXV.

### Essays and Cases.

ART. PAGE.

I.—Observations on Scarlet Fever. By Dr. Silas Reed, - - - - - - 8

II.—Remarks on Scarlet Fever. By Dr. George E. Conant, - - - - - - 23

III.—Notes of a Case in which Hydrophobia was prevented. By Landon C. Rives, M. D. - - 27

IV.—Observations on some of the uses and abuses of Purgatives. By Daniel Drake, M. D. - - 32

### Reviews and Bibliographical Notices.

Epidemic Cholera: An Eclectic, Miscellaneous and Clinical Review, - - - - - - 43

Mr. James Kennedy, - - - 44

Doctor Thomas Spencer, - - - - 50

—— Martin Paine, - - - 58

Mr. J. F. Fergus, - - - - 61

Doctor John F. Henry, - - - 66

—— Yates, - - - - - - 70

—— Sturm, - - - - 71

—— Stevens, - - - - - 72

—— Jackson, - - - - 78

—— Benjamin W. Dudley, - - - 80

In Lexington, - - - - 97

In Kentucky, - - - - 93

In Ohio—Contagion, - - - 96

In Cincinnati, - - - - 98

### Miscellaneous Intelligence.

ANALECTIC.

1. Prof. Ehrenberg's discoveries relative to the structure and functions of the Infusoria, - - - 105

2. Physiological investigations arising from the mechanical effects of pressure on the animal frame, - 116

CONTENTS.

3. Quantity of food taken by a person in health compared with the quantity of the different secretions during the same time, - - - - - - 120
4. Experiments on Hemlock and Henbane, - 122
5. Cold dash in Convulsions of Infants, - - 123
6. On the Excisions of Hæmorrhoidal Tumours, - 123
7. Treatment of Prolapsus Ani, - - - 131
8. Treatment of Cholera by Cold, as practised by Dr. Caspar, of Berlin, - - - - 133
9. Dr. Hancock on External Stimulants, - - 135
10. Clinical Observations on the Exhibition of Opium, in large doses, in certain cases of Disease. By Dr. W. Stokes, - - - - - - 139
11. Dr. Broussais' Opinion on the Use of Tartar Emetic in Pneumonia, - - - - - - 144
12. Cholera, great variety in the Treatment of, in Paris, 145

ANALYTICAL.

1. Chemical and Medical Qualities of the Apocynum Cannabinum, - - - - - - 146
2. Medicinal Properties of the Euphorbia Corollata, 147
3. Spontaneous poisonous changes in Meat and Bread, 149

ORIGINAL.

1. Our Enterprize, - - - - - - 151
2. The Epidemic, - - - - - 153
3. Measles and Mumps in Combination, - 154
4. Convulsions in Infants, - - - - 155
5. Animalcules in our hydrant water. - - 155
6. Neglect of Vaccination. Free Schools, - - 156
7. Inquiry into the Condition of the Medical College of Ohio, - - - - - - - 156
8. Western Graduates, - - - - 157
9. Popular Lectures on Anatomy and Surgery, - 157
10. Lithotomy, - - - - - - - 158
11. Medical Obituary, - - - - 158

Doctor Roane, - - - - - - 158
—— Pindell, - - - - - 159
—— Challen, - - - - - 159
—— Flanner, - - - - - 159
—— Richards, - - - - - 159
—— Heermann, - - - - 160

